# The influence of gender on outcomes following transcatheter aortic valve implantation

**DOI:** 10.3389/fcvm.2024.1417430

**Published:** 2024-07-17

**Authors:** Vittoria Lodo, Enrico G. Italiano, Luca Weltert, Edoardo Zingarelli, Chiara Perrucci, Claudio Pietropaolo, Gabriella Buono, Paolo Centofanti

**Affiliations:** ^1^ Department of Cardiac Surgery, Azienda Ospedaliera Ordine Mauriziano di Torino, Turin, Italy; ^2^ Division of Cardiac Surgery, Department of Cardiac, Thoracic, Vascular Sciences and Public Health, University of Padova, Padua, Italy; ^3^ Department of Cardiovascular Sciences, European Hospital, Rome, Italy; ^4^ Department of Cardiovascular Anesthesia and Intensive Care, Azienda Ospedaliera Ordine Mauriziano, Turin, Italy

**Keywords:** aortic stenosis, TAVI, transcatheter aortic valve implantation, sex differences, gender differences

## Abstract

**Objectives:**

This study aimed to compare gender-related differences in short- and long-term outcomes after transcatheter aortic valve implantation.

**Methods:**

Patients who underwent transcatheter aortic valve implantation (TAVI) for severe aortic stenosis (AS) from September 2017 to December 2022 were enrolled. The primary endpoint was 5-year all-cause mortality. The secondary endpoints were 30-day mortality and the incidence of post-procedural complication. Patients were separated according to gender before statistical analysis. To compare patients with similar baseline characteristics, we performed a propensity matching.

**Results:**

A total of 704 patients [females, 361 (51.3%); males, 343 (48.7%)] were enrolled. Compared to women, men had a higher incidence of smoking (40.5% vs. 14.7%, *p* < 0.001), diabetes (32.9% vs. 25.1%, *p* < 0.025), peripheral artery disease (35.8% vs. 18.3%, *p* < 0.001), and previous cardiac surgery (13.7% vs. 7.2%, *p* = 0.006) and a lower ejection fraction [56.6 (9.3) vs. 59.8 (7.5), *p* = 0.046]. Female patients were frailer at the time of the procedure [poor mobility rate, 26% vs. 11.7%, *p* < 0.001; CCI (Charlson comorbidity index) 2.4 (0.67) vs. 2.32 (0.63), *p* = 0.04]. Despite these different risk profiles, no significant differences were reported in terms of post-procedural outcomes and long-term survival. Propensity score matching resulted in a good match of 204 patients in each group (57.9% of the entire study population). In the matched cohort, men had a significantly higher incidence of new pacemaker implantation compared to women [33 (16.2%) vs. 18 (8.8%)]. The Kaplan–Meier 5-year survival estimate was 82.4% for women and 72.1% for men, *p* = 0.038.

**Conclusions:**

Female gender could be considered as a predictor of better outcomes after TAVI.

## Introduction

Female gender has been recognized as a risk factor for mortality and morbidity after cardiac surgery (1, 2). Indeed, despite advantages in cardiac surgery with new techniques and procedural innovations, improvements in outcomes have not been uniform across genders.

This has been largely demonstrated in coronary artery bypass grafting (CABG) surgery (3, 4) and surgical valve procedures (5, 6) where the burden of postoperative morbidity and mortality is significantly higher among women when compared to men.

However, the evidence base for the impact of gender upon transcatheter aortic valve implantation (TAVI) remains evolving. Some studies suggest improved outcomes among women (5–7) whereas other studies report results that are similar to men (8).

This study aims to retrospectively assess gender differences in terms of preoperative risk profile and short and long-term outcomes, in patients undergoing TAVI.

## Material and methods

The study was conducted in accordance with the ethical principles reported in the Declaration of Helsinki, and the study design was approved by the local Ethics Committee at the Mauriziano Hospital, Turin—Italy (protocol number 260-2022). Informed consent was obtained from all patients.

### Patient population and study design

From September 2017 to December 2022, 1,096 consecutive patients with a diagnosis of severe aortic stenosis (AS) were referred to our department. Among these, 704 patients underwent TAVI.

The criteria for TAVI implantation were based on the recommendations of the last European guidelines for the management of valvular heart disease (9).

Each patient was allocated to the most appropriate approach after an accurate multidisciplinary evaluation based on clinical history, blood tests, electrocardiogram, transthoracic echocardiography, computed tomography, and cardiac catheterization.

The main inclusion criteria were the diagnosis of severe aortic valve stenosis, with or without coronary disease requiring concomitant percutaneous coronary intervention (PCI).

For the purpose of the study, patients were separated according to gender (male and female) before statistical analysis.

The baseline characteristics such as age, body surface area (BSA), body mass index (BMI), hypertension, dyslipidemia, diabetes, smoke, previous cerebrovascular events, kidney function, peripheral artery disease (PAD), chronic obstructive pulmonary disease (COPD), poor mobility, previous cardiac surgery, history of heart failure, previous coronary disease, ejection fraction (EF), anatomic valvular area (AVA), mean transvalvular gradient, pulmonary hypertension, right bundle branch block (RBBB), and New York Heart Association (NYHA) class were evaluated in both groups.

The Charlson comorbidity index (CCI) and poor mobility were used to assess patient frailty (10).

Procedural risk profile was assessed using EuroSCORE II (11) and STS score (12).

Intraoperative data such as prosthesis type, need for concomitant valvuloplasty (pre/postimplantation), PCI or coronary ostia protection, and the access site were collected.

### Operative technique

The majority of patients underwent transfemoral TAVI under conscious sedation. Both surgical and percutaneous transfemoral access were performed. In the case of the percutaneous approach, the main vascular access puncture was performed under an ultrasound guide, and vascular access was closed using ProGlide (Abbott Vascular, CA, USA) or Manta (Teleflex, PA, USA) device.

Alternative accesses such as trans-subclavian, trans-carotid, and transapical were used only when the transfemoral approach was not feasible.

Both new-generation self-expandable and balloon-expandable prostheses were implanted. The choice of prosthesis size and vascular access was based on pre-procedural computed tomography.

Balloon aortic valvuloplasty, before and/or after TAVI, was performed at the operator’s discretion.

When required, ventricular pacing was performed using a ventricular temporary pacemaker.

Cardiac catheterization was performed mainly during the procedure, and if required, a concomitant PCI was performed. In all cases, a complete percutaneous revascularization was performed.

If required, coronary protection was achieved as follows. Via the radial artery, a coronary wire was advanced in the targeted coronary, and an undeployed coronary balloon or stent was positioned in the coronary prior to the beginning of valve deployment in preparation for emergent usage following TAVI.

### Outcomes

The primary outcome assessed for the present study was 5-year all-cause mortality.

The secondary endpoints were 30-day mortality, mortality from cardiac causes, postoperative acute kidney injury (AKI), neurological events, new pacemaker (PM) implantation, new-onset atrial fibrillation (AF) and left bundle branch block (LBBB), inotropic support, paravalvular leak (PVL), prosthesis mean gradient, access site complications, emergency surgical conversion, and in-hospital length of stay.

AKI’s definition was based on Kidney Disease: Improving Global Outcome (KDIGO) criteria (13).

All the patients received a follow-up visit at 3 months after the procedure and a follow-up phone call every year.

The follow-up was completed on 31 December 2023.

### Statistical analysis

The continuous variables data were presented as mean and standard deviation (SD), whereas the categorical variables data were expressed as frequency and percentages.

In univariate analysis, the continuous variables were compared using a *t*-test or Wilcoxon–Mann–Whitney test according to distribution type, while the categorical variables were compared using a chi-squared test or Fisher’s exact test.

Five-year all-cause mortality was assessed and reported using the Kaplan–Meier method, and the survival curves were compared using the log-rank test (Mantel–Cox).

To reduce possible differences between the two study groups, a matched analysis using propensity score was performed. Propensity matching was performed by running a logistic binary regression, with gender as the dependent variable, the probability of the regression was stored and used as a matching score by best neighbor matching. The overall efficacy of the match method was then tested rerunning the logistic regression and verifying that no variables had significant differences.

Impactful variables on univariate analysis in terms of both mortality and morbidity were included to avoid heavy preconditioners being unbalanced in the caseload.

The *a priori* selected variables were as follows: diabetes, smoke, PAD, history of cerebrovascular events, previous cardiac surgery, poor mobility, EF, and NYHA class.

All *p*-values were two-sided, and a *p*-value <0.05 was considered statistically significant. All analyses were performed with SPSS 26.0 (IBM, Chicago, USA).

## Results

### Unmatched patient cohort

From September 2017 to December 2022, 704 consecutive patients with a diagnosis of severe aortic valve stenosis underwent TAVI at Mauriziano Hospital in Italy and were enrolled in the present analysis. A total of 343 males were compared to 361 females.

The baseline characteristics and comorbidities are reported in Table 1.

**Table 1:** Baseline characteristics and comorbidities of the unmatched cohort.

Variables	M (*n* = 343)	F (*n* = 361)	*p*-value
Age, mean (SD), years	82.14 (5.01)	82.43 (5.06)	0.448
BMI, mean (SD), kg/m^2^	26.34(4.22)	26.14 (5.79)	0.600
BSA, mean (SD), m^2^	1.8 (0.43)	1.68 (0.38)	0.061
Hypertension, *n* (%)	315 (91.8%)	328 (90.9%)	0.689
Diabetes, *n* (%)	113 (32.9%)	91 (25.1%)	**0.025**
Dyslipidemia, *n* (%)	184 (53.6%)	184 (51%)	0.497
Smoke, *n* (%)	139 (40.5%)	53 (14.7%)	**<0.001**
COPD, *n* (%)	70 (20.4%)	70 (19.4%)	0.777
PAD, *n* (%)	121 (35.8%)	66 (18.3%)	**<0.001**
History of cerebrovascular events, *n* (%)	20 (5.8%)	10 (2.7%)	0.061
eGFR, mean (SD), ml/min	65 (21)	60 (18)	0.467
RRT, *n* (%)	9 (2.7%)	6 (1.7%)	0.439
Poor mobility, *n* (%)	40 (11.7%)	94 (26%)	**<0.001**
CCI, *n* (%)	2.32 (0.63)	2.4 (0.67)	**0.04**
History of heart failure, *n* (%)	103 (30%)	101 (28%)	0.562
History of coronary disease, *n* (%)	40 (11.7%)	36 (9.9%)	0.113
RBBB, *n* (%)	23 (6.7%)	22 (6.1%)	0.435
Redo surgery, *n* (%)	47 (13.7%)	26 (7.2%)	**0**.**006**
EF, mean (SD)	56.6 (9.3)	59.8 (7.5)	**0**.**046**
Mean gradient, mean (SD), mmHg	53.2 (11.4)	51.4 (12.6)	0.635
AVA, mean (SD), cm^2^	0.71 (0.31)	0.69 (0.43)	0.735
LF-LG AS, *n* (%)	13 (3.79%)	9 (2.49%)	0.114
Paradoxical LF-LG AS, *n* (%)	8 (2.33%)	11 (3.04%)	0.224
PAPs>55 mmHg, *n* (%)	16 (4.7%)	25 (6.9%)	0.260
NYHA III–IV, *n* (%)	152 (44.3%)	185 (51.2%)	0.070
EuroSCORE II, mean (SD)	4.38 (4.42)	4.81 (4.75)	0.194
STS score, mean (SD)	3.81 (3.5)	3.98 (3.6)	0.785

SD, standard deviation; BMI, body mass index; BSA, body surface area; COPD, chronic obstructive pulmonary disease; PAD, peripheral artery disease; eGFR, estimated glomerular filtration rate; RRT, renal replacement therapy; CCS, Charlson comorbidity index; RBBB, right bundle branch block; EF, ejection fraction; AVA, anatomic valvular area; LF-LG, low-flow low-gradient; AS, aortic stenosis; PAPs, pulmonary artery systolic pressure; NYHA, New York Heart Association.

Bold values represent statistical significant differences between the two study groups.

Men had a higher incidence of smoking (40.5% vs. 14.7%, *p* < 0.001), diabetes (32.9% vs. 25.1%, *p* < 0.025), PAD (35.8% vs. 18.3%, *p* < 0.001), and previous cardiac surgery (13.7% vs. 7.2%, *p* = 0.006) and a lower EF [56.6 (9.3) vs. 59.8 (7.5), *p* = 0.046] when compared to women.

Female patients were frailer at the time of the procedure (poor mobility rate 26% vs. 11.7%, *p* < 0.001; CCI 2.4 (0.67) vs. 2.32 (0.63), *p* = 0.04].

Intraoperative data and post-procedural outcomes are reported in Table 2.

**Table 2:** Intraoperative data and post-procedural outcomes of the unmatched cohort.

Variables	M (*n* = 343)	F (*n* = 361)	*p*-value
Self-expandable prosthesis, *n* (%)	182 (53.1%)	256 (70.9%)	**<0.001**
Balloon-expandable prosthesis, *n* (%)	161 (46.9%)	105 (29.1%)	**<0.001**
Transfemoral access, *n* (%)	321 (93.6%)	347 (96.1%)	0.170
• Percutaneous access	258 (80.4%)	277 (79.8%)	0.235
• Surgical access	63 (19.6%)	70 (20.2%)	0.235
Valvuloplasty (pre/post implantation), *n* (%)	102 (29.7%)	162 (44.9%)	**<0.001**
Concomitant PCI, *n* (%)	36 (10.5%)	30 (8.3%)	0.366
• Proximal CAD^a^	24 (66.7%)	21 (70%)	0.435
• Complete revascularization	36 (100%)	30 (100%)	–
Coronary ostia protection, *n* (%)	12 (3.5%)	15 (4.1%)	0.654
AKI, *n* (%)	20 (5.8%)	24 (6.6%)	0.756
Stroke, *n* (%)	7 (2%)	5 (1.4%)	0.569
Inotropic support, *n* (%)	5 (1.5%)	9 (2.5%)	0.421
New-onset LBBB^b^, *n* (%)	69 (20.1%)	81 (22.4%)	0.463
New-onset AF^b^, *n* (%)	5 (1.5%)	11 (3%)	0.207
PM implantation, *n* (%)	45 (13.1%)	37 (10.2%)	0.242
Access site complication, *n* (%)	30 (8.7%)	37 (10.2%)	0.523
Emergency surgical conversion, *n* (%)	2 (0.6%)	4 (1.1%)	0.687
PVL (at least mild–moderate), *n* (%)	17 (4.9%)	21 (5.8%)	0.622
Mean gradient, mean (SD), mmHg	8.64 (3.88)	9.35 (5.45)	0.069
In-hospital stay, mean (SD), days	5.91 (8.86)	5.53 (3.24)	0.450
30-day mortality, n	2 (0.9%)	5 (1.4%)	0.726
5-year all-cause mortality, *n* (%)	85 (24.78%)	89 (24.65%)	0.61
5-year mortality from cardiac causes, *n* (%)	56 (16.32%)	56 (14.95%)	0.635

PCI, percutaneous coronary intervention; CAD, coronary artery disease; AKI, acute kidney injury; LBBB, left bundle branch block; AF, atrial fibrillation; PM, pacemaker; PVL, paravalvular leak.

^a^ Proximal CAD refers to a CAD involving the left main or the proximal part of the left anterior descending.

^b^ For LBBB and AF at-discharge percentages, the denominator was the number of patients who developed post-procedural LBBB and AF in each group.

Bold values represent statistical significant differences between the two study groups.

No significant differences between males and females were reported in terms of percutaneous revascularization (10.5% vs. 8.3, *p* = 0.366) and the need for coronary ostia protection (3.5% vs. 4.1%, *p* = 0.654).

Women were more likely to receive a self-expandable prosthesis (70.9% vs. 53.1%, *p* < 0.001) and a concomitant valvuloplasty (44.9% vs. 29.7%, *p* < 0.001) when compared to men.

The incidence of death from any cause at 30 days was 0.9% (2/343) in male patients and 1.4% (3/361) in female patients (*p* = 0.076).

No significant differences between males and females were recorded in terms of neurological (2% vs. 1.4%, *p* = 0.569) and nephrological complications (5.8% vs. 6.6%, *p* = 0.756), access site complications (8.7% vs. 10.2%, *p* = 0.523), need for inotropic support (1.5% vs. 2.5%, *p* = 0.421), emergency surgical conversion (0.6% vs. 1.1%, *p* = 0.687), conduction abnormalities (AF, 1.5% vs. 3%, *p* = 0.207; LBBB, 20.1% vs. 22.4%, *p* = 0.463; new PM implantation, 13.1% vs. 10.2%, *p* = 0.242), and in-hospital stay [5.91 (8.86) vs. 5.53 (3.24) days, *p* = 0.450].

Regarding at-discharge echocardiography, no significant differences were reported in terms of PVL (4.9% vs. 5.8%, *p* = 0.622) and mean prosthesis gradient [8.64 (3.88) vs. 9.35 (5.45) mmHg, *p* = 0.069].

The mean follow-up period in the entire study population was 1,317 ± 493 days, whereas the mean follow-up period in the male and female cohorts was 1,298 ± 502 days and 1,322 ± 514 days, respectively.

**Figure 1:**
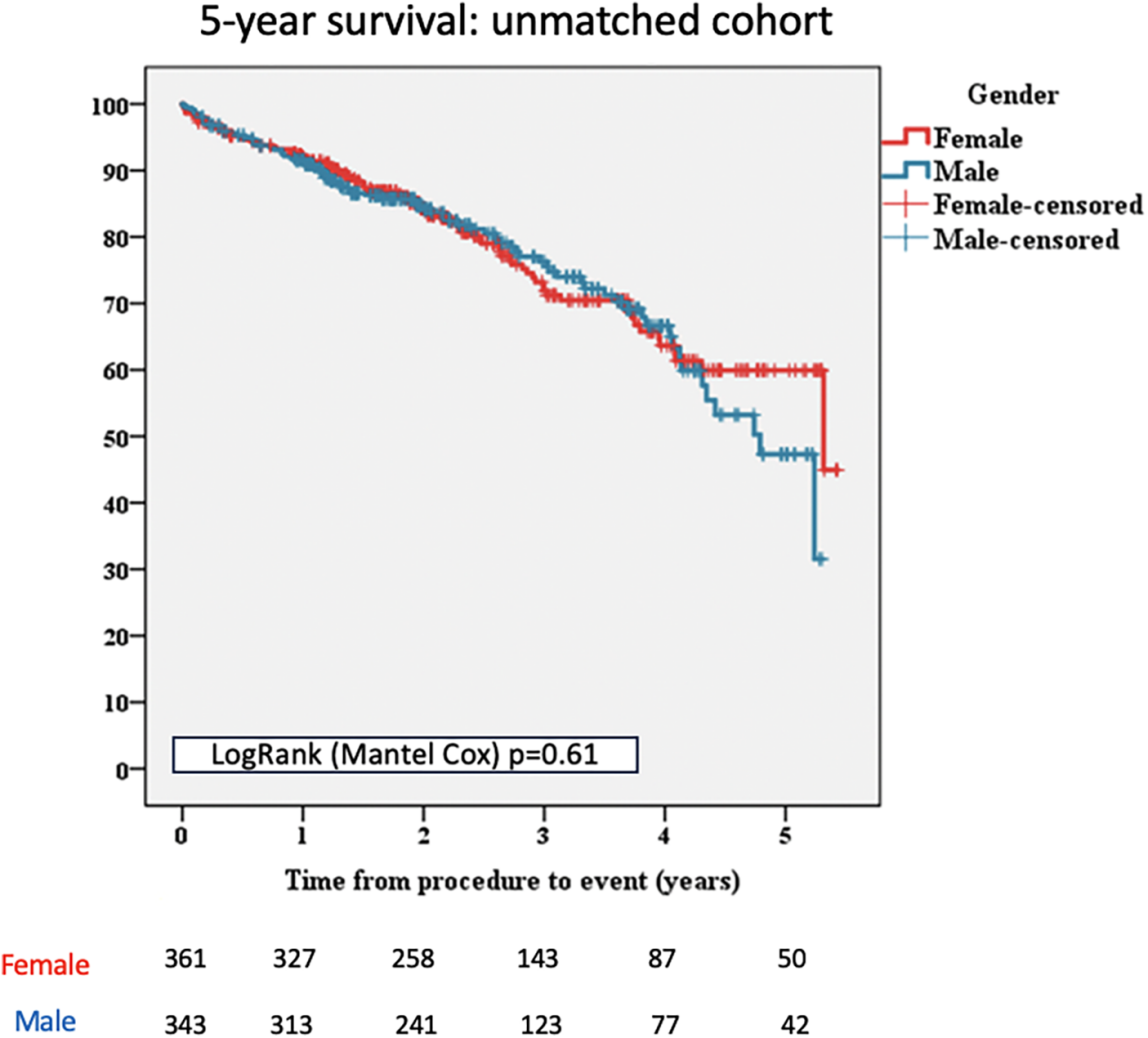
Kaplan–Meier survival curves for female and male patients, unmatched cohort. TAVI, transcatheter aortic valve implantation.

No significant differences were reported regarding 5-year all-cause mortality when compared to women and men [*n* = 89/361 (24.65%) vs. *n* = 85/343 (24.78%), *p* = 0.61; Figure 1].

Furthermore, no significant differences, between males and females, were reported in terms of death from cardiac causes [*n* = 56/343 (16.32%) vs. *n* = 56/361 (15.51%), *p* = 0.635; Table 2].

### Matched patient cohort

Propensity score matching of 361 female patients and 343 male patients resulted in a good match of 204 patients in each group (57.9% of the entire study population).

Table 3 shows the baseline characteristics and comorbidities of the matched groups.

**Table 3:** Baseline characteristics and comorbidities of the matched cohort.

Variables	M (*n* = 204)	F (*n* = 204)	*p*-value
Age, mean (SD), years	82.5 (5.0)	82.1 (4.8)	0.377
BMI, mean (SD), kg/m^2^	26.4 (4.3)	26.1 (5.7)	0.530
BSA, mean (SD), m^2^	1.77 (0.31)	1.69 (0.43)	0.073
Hypertension, *n* (%)	183 (89.7%)	186 (91.2%)	0.737
Diabetes, *n* (%)	55 (27%)	59 (28.9%)	0.741
Dyslipidemia, *n* (%)	97 (47.5%)	107 (52.5%)	0.373
Smoke, *n* (%)	52 (25.5%)	50 (24.5%)	0.909
COPD, *n* (%)	34 (16.7%)	42 (20.6%)	0.373
PAD, *n* (%)	50 (24.5%)	55 (27%)	0.651
History of cerebrovascular events, *n* (%)	3 (1.5%)	7 (3.4%)	0.338
eGFR, mean (SD), ml/min	62 (16)	61 (15)	0.786
RRT, *n* (%)	8 (4.1%)	3 (1.5%)	0.139
Poor mobility, *n* (%)	27 (13.2%)	31 (15.2%)	0.671
CCI, mean, (SD)	2.34 (0.64)	2.36 (0.65)	0.065
History of heart failure, *n* (%)	58 (28.4%)	54 (26.5%)	0.739
History of coronary disease, *n* (%)	30 (14.7%)	25 (12.2%)	0.235
RBBB, *n* (%)	14 (6.86%)	12 (5.88%)	0.145
Redo surgery, *n* (%)	23 (11.3%)	22 (10.8%)	1.00
EF, mean (SD)	59.7 (8.8)	59.9 (8.6)	0.778
Mean gradient, mean (SD), mmHg	52.3 (10.5)	50.6 (13.6)	0.538
AVA, mean (SD), cm^2^	0.69 (0.36)	0.67 (0.38)	0.355
LF-LG AS, *n* (%)	5 (2.45%)	6 (2.94%)	0.546
Paradoxical LF-LG AS, *n* (%)	1 (0.49%)	2 (0.98%)	1,000
PAPs>55 mmHg, *n* (%)	6 (2.9%)	15 (7.4%)	0.071
NYHA III–IV, *n* (%)	80 (39.2%)	84 (41.2%)	0.762
EuroSCORE II, mean (SD)	4.40 (4.52)	4.74 (4.68)	0.173
STS score, mean (SD)	3.78 (3.2)	3.89 (3.5)	0.823

SD, standard deviation; BMI, body mass index; BSA, body surface area; COPD, chronic obstructive pulmonary disease; PAD, peripheral artery disease; eGFR, estimated glomerular filtration rate; RRT, renal replacement therapy; CCI, Charlson comorbidity index; RBBB, right bundle branch block; EF, ejection fraction; AVA, anatomic valvular area; LF-LG, low-flow low-gradient; AS, aortic stenosis; PAPs, pulmonary artery systolic pressure NYHA, New York Heart Association.

After performing the propensity score matching, there was an excellent matching of all the variables between the 204 paired patients in each group, including for important prognostic markers such as age, cardiovascular risk profile, frailty, echocardiographic data, previous cardiac operations, and EuroSCORE II.

Intraoperative data and post-procedural outcomes of the matched patient cohort are reported in Table 4.

**Table 4:** Intraoperative data and post-procedural outcomes of the matched cohort.

Variables	M (*n* = 204)	F (*n* = 204)	*p*-value
Self-expandable prosthesis, *n* (%)	109 (53.4%)	147 (72.1%)	<0.001
Balloon-expandable prosthesis, *n* (%)	95 (46.6%)	57 (27.9%)	<0.001
Transfemoral access, *n* (%)	195 (95.6%)	198 (97.1%)	0.600
• Percutaneous access	161 (82.6%)	165 (83.3%)	0.689
• Surgical access	34 (17.4%)	33 (16.7%)	0.689
Valvuloplasty (pre-/postimplantation), *n* (%)	58 (28.4%)	91 (44.6%)	<0.001
Concomitant PCI, *n* (%)	26 (12.7%)	21 (10.3%)	0.535
• Proximal CAD^a^	19 (73.1%)	15 (71.4%)	0.502
• Complete revascularization	26 (100%)	21 (100%)	–
Coronary ostia protection, *n* (%)	6 (2.9%)	5 (2.4%)	0.872
AKI, *n* (%)	13 (6.4%)	15 (7.4%)	0.845
Stroke, *n* (%)	6 (2.9%)	5 (2.4%)	1,000
Inotropic support, *n* (%)	4 (1.9%)	6 (2.9%)	0.751
New-onset LBBB^b^, *n* (%)	38 (18.6%)	50 (24.5%)	0.185
New-onset AF^b^, *n* (%)	4 (2%)	6 (2.9%)	0.751
PM implantation, *n* (%)	33 (16.2%)	18 (8.8%)	**0.035**
Access site complication, *n* (%)	19 (9.3%)	21 (10.3%)	0.868
Emergency surgical conversion, *n* (%)	1 (0.5%)	2 (1.0%)	1,000
PVL (at least mild–moderate), *n* (%)	7 (3.4%)	15 (7.4%)	0.123
Mean gradient, mean (SD), mmHg	8.7 (3.8)	9.6 (5.9)	0.083
In-hospital stay, mean (SD), days	6.6 (11)	5.4 (3.4)	0.127
30-day mortality, *n*	3 (1.5%)	3 (1.5%)	0.996
5-year all-cause mortality, *n* (%)	57 (27.9%)	36 (17.6%)	0.038
5-year mortality from cardiac causes, *n* (%)	43 (21.8%)	26 (12.7%)	0.042

PCI, percutaneous coronary intervention; CAD, coronary artery disease; AKI, acute kidney injury; LBBB, left bundle branch block; AF, atrial fibrillation; PM, pacemaker; PVL, paravalvular leak.

^a^ Proximal CAD refers to a CAD involving the left main or the proximal part of the left anterior descending.

^b^ For LBBB and AF at-discharge percentages, the denominator was the number of patients who developed post-procedural LBBB and AF in each group.

Bold values represent statistical significant differences between the two study groups.

Intraoperative data did not show any changes after propensity score matching. Women were still more likely to receive a self-expandable prosthesis (72.1% vs. 53.4%, *p* < 0.001) with concomitant valvuloplasty (41.6% vs. 28.4%, *p* < 0.001), and no significant differences were reported in terms of number of patients requiring concomitant PCI (12.7% vs. 10.3%, *p* = 0.535) and coronary ostia protection (2.9% vs. 2.4%, *p* = 0.872).

There was further no difference between 30-day mortality (1.5% vs. 1.5%, *p* = 0.006), in-hospital stay [6.6 (11) vs. 5.4 (3.4) days, *p* = 0.127), neurological (2.9% vs. 2.4%, *p* = 1.000) and nephrological (6.4% vs. 7.4%, *p* = 0.845) complications, new-onset LBBB (18.6% vs. 24.5%, *p* = 0.185) and AF (2% vs. 2.4%, *p* = 0.751), access site complications (9.3% vs. 10.3%, *p* = 0.868), emergency surgical conversion (0.5% vs. 1%, *p* = 1.000), PVL (3.4% vs. 7.4%, *p* = 0.123), and mean gradient [8.7 (3.8) vs. 9.6 (5.9) mmHg, *p* = 0.083], for female and male patients.

A total of 33 (16.2%) male patients required a new permanent PM implantation, while 18 (8.8%) needed a PM implantation among female patients (*p* = 0.035).

The mean follow-up period in the entire matched cohort was 1,322 ± 518 days, whereas the mean follow-up period in the male and female matched cohorts was 1,301 ± 512 days and 1,330 ± 502 days, respectively.

**Figure 2:**
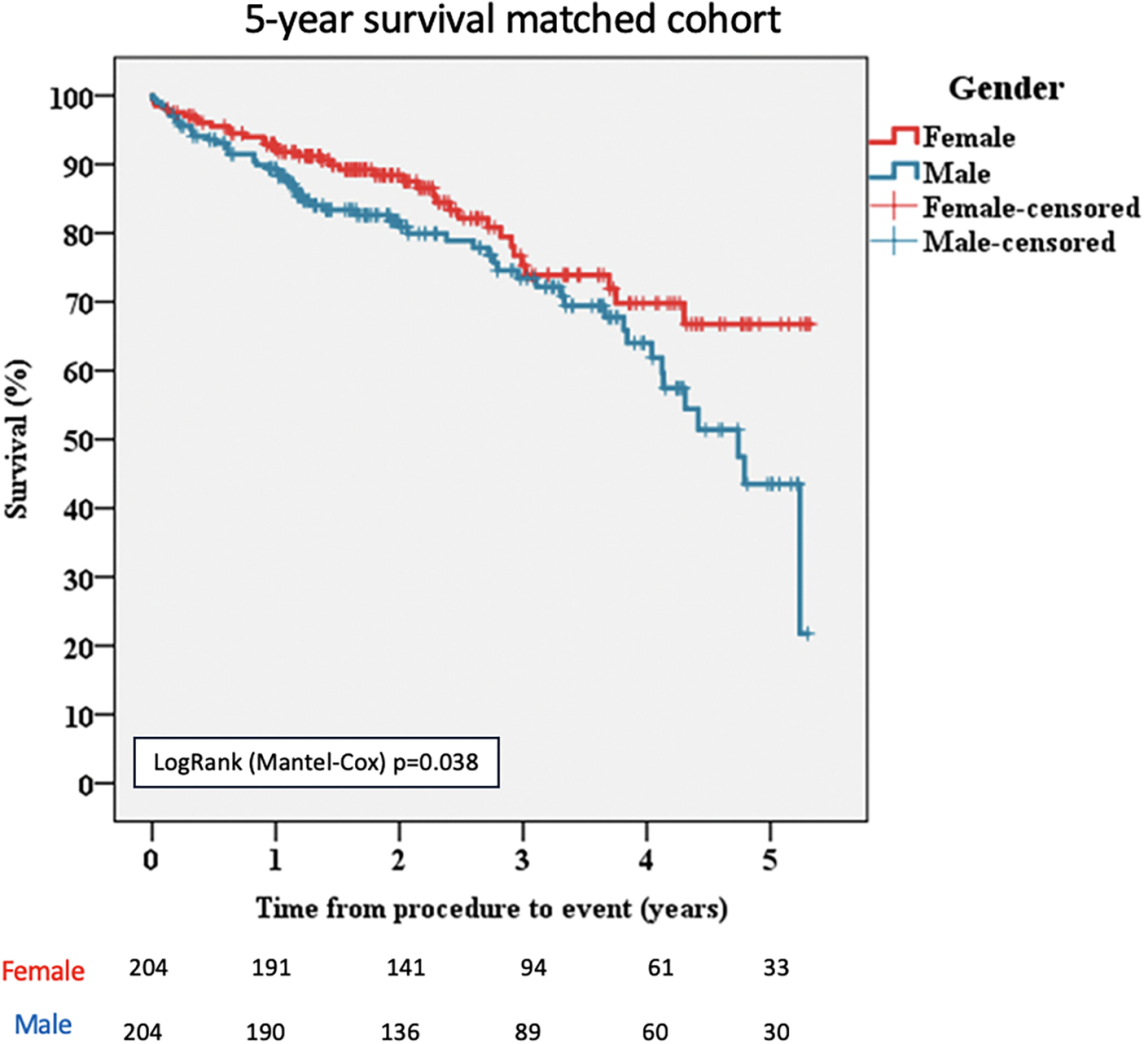
Kaplan–Meier survival curves for female and male patients, matched cohort. TAVI, transcatheter aortic valve implantation.

In the propensity-matched population, during the 5-year follow-up, there were 36 deaths (17.64%) among women compared with 57 deaths (27.94%) among men (*p* = 0.038; Figure 2).

Death from cardiac causes was significantly higher in men than in women [*n* = 43/204 (21.08%) vs. *n* = 26/204 (12.74%), *p* = 0.042; Table 4].

## Discussion

In this single-center retrospective study, evaluating sex-related differences in post-procedural outcomes and 5-year survival in patients undergoing TAVI, we found that there were no significant differences in the unmatched population.

However, after propensity score matching women had a significantly higher 5-year survival and a lower incidence of new permanent PM implantation when compared to men. Moreover, women showed a lower rate of death from cardiac causes.

The prevalence of severe AS requiring intervention is constantly increasing as the size of the elderly population increases (14, 15). Consequently, the interest in the most appropriate approach for each patient has rapidly grown, and particularly attention has been given to gender differences.

Female gender has been commonly considered a risk factor in cardiac surgery (2), and a large number of studies have suggested that surgical aortic valve replacement (SAVR) could represent a greater risk for women than for men.

The initial indication of worse outcomes for women originated from the PARTNER trial where female patients treated with SAVR showed a lower 1-year survival when compared to TAVI (16).

In their meta-analysis, Panoulas et al. (17) demonstrated that women undergoing TAVI had significantly lower mortality compared to those undergoing SAVR (31% reduction at 1 year and 26% reduction at 5 years). A similar difference was not found in the male population.

Many explanations for these results have been provided. Compared with men, women tend to present later in their disease process, are usually older, and have poorer preoperative risk profiles and a more challenging anatomy (5, 18).

As reported by Sannino et al. (19, 20), the absence of differential cutoff values in male and female patients to identify valvular heart disease-related adverse cardiac remodeling or to define the optimal timing for intervention could be at the base of late referral and worse outcomes of female patients.

According to their higher procedural risk, women are more often candidates for the transcatheter approach, while men are more likely to receive surgical treatment.

As a consequence, in contrast to most trials in the cardiovascular field, in which women have been usually underrepresented, the portion of female patients in TAVI studies is at least 50%, which allows an accurate gender comparison (21).

Nonetheless, the influence of gender on outcomes following cardiac surgery is still controversial. Some studies have shown a higher mortality in men (22), while others have not identified significant differences between genders (23, 24).

Our study population was similar to those reported in the literature: female patients were more represented and frailer when compared to male patients, while men had a higher cardiovascular risk profile, higher rate of previous cardiac operations, and lower left ventricle function than female patients. However, the operative risk score did not present a significant difference between the two groups (EuroSCORE II, 4.38 ± 4.42 vs. 4.81 ± 4.75, *p* = 0.194; STS score, 3.81 ± 3.5 vs. 3.98 ± 3.6).

Despite the abovementioned preoperative characteristics, we did not find significant differences in terms of post-procedural outcome and long-term follow-up.

We can suppose that the higher rate of pre-procedural comorbidities in male patients might have been balanced by the major preoperative frailty in female patients.

Outcomes significantly changed after propensity score matching. Men showed a significantly higher incidence of PM implantation (16.2% vs. 8.8%, *p* = 0.035), despite they were more likely to receive a balloon-expandable prosthesis that is associated with a lower incidence of PM implantation when compared to self-expandable prosthesis (25). Furthermore, men did not present a higher incidence of preoperative RBBB, which is a well-known risk factor for post-procedural PM implantation. We assumed that the higher incidence of PM implantation in male patients may be related to a different degree and distribution of valve calcifications in male vs. female patients.

Our result is corroborated by the meta-analysis of Ravaux et al. (26). Based on 46 studies reporting information about the impact of patient sex on PM implantation after TAVI, they demonstrated that female gender is associated with a lower risk of post-procedural PM implantation when compared to men.

Regarding long-term follow-up, 5-year survival was significantly higher in women (82.4% vs. 72.1%; *p* = 0.038).

This result is corroborated by several studies.

A large report from the ACC/TVT Registry evaluated gender differences among 11.808 patients who underwent TAVI for severe AS. No differences were reported in terms of in-hospital mortality, but female patients had significantly lower 1-year mortality than men (adjusted hazard ratio, 0.73; 95% IC, 0.63–0.85; *p* < 0.001) (7).

A meta-analysis by O’Connor et al. showed that women had similar mortality to men at 30 days but had significantly better long-term survival (adjusted hazard ratio, 0.79; 95% CI, 0.73–0.86; *p* < 0.001) (27).

Similarly, Saad et al. reported a lower 1-year mortality for female patients when compared to men (28).

The reason for the higher survival rate in female patients undergoing TAVI is still not completely clear.

First female patients have a longer life expectancy. Nevertheless, this cannot be the only explanation for the better long-term survival of female patients. In fact, in the unmatched cohort, despite the higher cardiovascular risk profile of the male patients and the higher women’s life expectancy, we did not find significant differences in terms of long-term outcomes.

On the other hand, women were frailer at the time of the procedure. Female patients showed a significantly higher CCI and had a significantly higher incidence of poor mobility, which can be considered a further indicator of patient frailty (29).

The higher frailty is the only unfavorable pre-procedural variable, which is more represented in female than in male patients and, interestingly, when its distribution is balanced between the two groups women showed a significantly higher long-term survival.

Our finding confirms that frailty is associated with an increased risk for adverse outcomes independent of age or other concomitant comorbidities (30).

In the matched cohort, male patients had a higher incidence of new PM implantation, which leads to mechanical dyssynchrony and may decrease ventricular function, with a negative impact on long-term survival and a higher risk of death from cardiac causes (31).

Finally, despite several studies reporting a higher incidence of vascular access complications and life-threatening bleeding among women (8), in our study, we did not report significant differences between the two groups.

These findings stress that an accurate choice of the access site, based on pre-procedural computed tomography, is mandatory to achieve the same results in female and male patients.

The single-center non-randomized study design is the main limitation. The results herein presented are specific to a single center and may not be easily applied across all hospital and geographic settings.

## Conclusion

Despite technical advantages, women showed a higher mortality and morbidity after cardiac surgery compared to men. However, transcatheter procedures seem not to follow this rule.

In our entire study population, there were no significant differences in terms of post-procedural outcomes and long-term survival, while in the propensity-matched subset of patients, men showed a significantly higher need for new PM implantation, a lower 5-year survival, and a higher rate of death from cardiac causes compared to women.

To conclude, the female gender could be recognized as a predictor of better outcomes after TAVI.

As a consequence, patient gender should be taken into deep consideration to allocate patients with severe aortic valve stenosis to the most appropriate approach.

## Data Availability

The raw data supporting the conclusions of this article will be made available by the authors, without undue reservation.
